# Reduced Risk-Taking following Disruption of the Intraparietal Sulcus

**DOI:** 10.3389/fnins.2016.00588

**Published:** 2016-12-23

**Authors:** Christopher G. Coutlee, Anastasia Kiyonaga, Franziska M. Korb, Scott A. Huettel, Tobias Egner

**Affiliations:** Department of Psychology and Neuroscience, Center for Cognitive Neuroscience, Duke UniversityDurham, NC, USA

**Keywords:** risk, ambiguity, uncertainty, neuroeconomics, intraparietal sulcus, TMS

## Abstract

Decision makers frequently encounter opportunities to pursue great gains—assuming they are willing to accept greater risks. Previous neuroimaging studies have shown that activity in the intraparietal sulcus (IPS) and the inferior frontal junction (IFJ) are associated with individual preferences for economic risk (“known unknowns,” e.g., a 50% chance of winning $5) and ambiguity (“unknown unknowns,” e.g., an unknown chance of winning $5), respectively. Whether processing in these regions causally enables risk-taking for individual decisions, however, remains unknown. To examine this question, we assessed the decision to engage in risk-taking after disrupting neural processing in the IPS and IFJ of healthy human participants using repetitive transcranial magnetic stimulation. While stimulation of the IFJ resulted in general slowing of decision times, disrupting neural processing within the IPS selectively suppressed risk-taking, biasing choices toward certain options featuring both lower risks and lower expected rewards. Our results are the first to demonstrate the necessity of intact IPS function for choosing uncertain outcomes when faced with calculable risks and rewards. Engagement of IPS during decision making may support a willingness to accept uncertain outcomes for a chance to obtain greater gains.

## Introduction

Decision making is often characterized by the need to make difficult tradeoffs between uncertain risks and rewards. Although excessive risk-seeking can be problematic (Yates, 1992), investors and economists have also recognized that obtaining greater rewards often requires accepting greater risk (Markowitz, 1952). An abundance of caution can sometimes endanger long-term financial goals such as retirement and homeownership (Bajtelsmit and VanDerhei, 1997), or lead to missed opportunities, such as when a promising job offer is turned down because it requires relocating to an unfamiliar city. Decision makers thus stand to benefit from control mechanisms capable of calibrating risk-taking behavior based on existing risk preferences as well as situational risks and benefits.

In the current investigation, we examine the contributions of two brain regions key to such flexible behavioral control during uncertain decisions: the intraparietal sulcus (IPS) and the inferior frontal junction (IFJ). Traditional (Mohr et al., 2010) and large-scale automated (Yarkoni et al., 2011) metanalyses of neuroimaging studies demonstrate consistent activation within the IPS and IFJ during uncertain decision making. Activation within these two regions has been found to scale with the degree of uncertainty as information is accumulated toward a decision (Huettel et al., 2005) and reflects outcome uncertainty in a manner dissociable from other choice-related processes (Bach et al., 2009). Information represented within the IFJ and IPS prior to an uncertain decision is also predictive of subsequent decisions to engage in risk-taking (Helfinstein et al., 2014).

The IPS and IFJ show evidence of differential sensitivity to two important forms of uncertainty: economic risk (“known unknowns,” e.g., a 50% chance of winning $5) and ambiguity (“unknown unknowns,” i.e., an unknown chance of winning $5; Knight, 1921; Ellsberg, 1961; Camerer and Weber, 1992). IPS activation is enhanced for risky choices relative to intertemporal choices (Weber and Huettel, 2008), with this activation preferentially tracking risky subjective value (Peters and Büchel, 2009). Similarly, neuronal activity measured in the non-human primate analog of IPS represents the relative subjective value of risky choices (Dorris and Glimcher, 2004). The IFJ, by contrast, is robustly active during ambiguous decision making (Huettel et al., 2006) and in response to ambiguous aversive cues (Bach et al., 2009), with individual differences in responses to ambiguity predicting behavioral ambiguity aversion (Bach et al., 2011). In an fMRI study directly comparing neural processing of risk and ambiguity, Huettel and colleagues identified a double-dissociation, with greater IPS activation predicting an increased acceptance of risk, and greater IFJ activation predicting an increased acceptance of ambiguity (Huettel et al., 2006). Such results thus strongly implicate the IPS and IFJ in uncertain decision making, and suggest that these regions may differentially represent uncertainty preferences during risky and ambiguous choices. Whether intact processing in these regions is actually required to engage in risk-taking, however, remains unknown.

**Figure 1:**
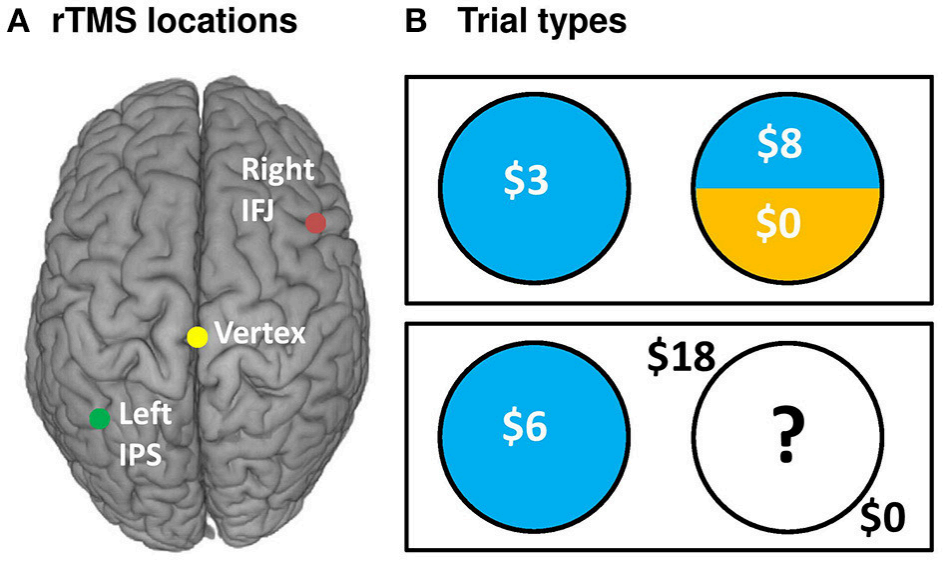
**rTMS targets and task design**. **(A)** rTMS was applied to three targets: the left IPS (−36, −57, 50), right IFJ (39, 16, 33), and between the cerebral hemispheres at vertex (0, −28, 90) as an active control. Targets were based on group level fMRI contrasts from our previous investigation of risk and ambiguity preferences (Huettel et al., 2006), and were located within each participant using their structural MRI scan from an earlier study. **(B)** Participants chose between a certain option (known outcome, left circles) and either a risky option (top right) or an ambiguous option (bottom right).

To address this question, we conducted an experiment in which participants engaged in risk-taking for monetary rewards while we manipulated both the type of uncertainty they faced—and critically—the integrity of neural processing within the IPS and IFJ. We applied MRI-guided 1-Hz repetitive transcranial magnetic stimulation (rTMS)—thought to inhibit (Chen et al., 1997) or disrupt (Harris et al., 2008) neural processing—over the IPS, IFJ, and a vertex control site in a counterbalanced within-subjects design (Figure 1A). After each rTMS treatment, we examined participants' risk-taking behavior (choices and response times) across a series of decision trials. Each decision pitted a certain but small reward against an uncertain reward (Figure 1B), with uncertainty being either “risky” (a known 25, 50, or 75% chance of reward) or “ambiguous” (unknown chance of reward). The expected value of the uncertain option was varied to offer a premium over the value of the certain option in a majority of trials, thereby incentivizing risk-taking to various degrees. Based on the previous findings discussed above, we made two independent predictions: First, relative to control rTMS, IPS rTMS would interfere with uncertain decision-making for risky decision trials; second, IFJ rTMS would likewise disrupt decision processes for ambiguous trials. We analyzed the effects of rTMS location on choices and response times for these decisions using a multilevel mixed-models approach for repeated measures, treating both decisions and participants as random (as opposed to fixed) effects. Our design thus allowed us to draw causal inferences regarding the importance of IPS and IFJ to decision making under both risk and ambiguity.

## Materials and methods

### Participants

Fifteen right-handed adult participants (mean age 27.43, range 19–43; six female) reported no history of psychiatric or neurological disorders and passed MRI and TMS safety screenings (Rossi et al., 2009). Written informed consent was obtained from each participant prior to each study session. Participants received $80, plus additional payments based on task decisions. Procedures were reviewed and approved by the Duke University institutional review board.

### Procedure overview

Participants completed separate 1-h MRI and 3-h rTMS visits. Anatomical MRI images were used for personalized neuro-navigated targeting during the rTMS study. Prior to receiving rTMS, participants were screened, familiarized with the study equipment, and trained on the decision-making task. Participants then completed a full (165 trials, paid) practice run of the task.

We conducted a within-subjects experiment in which each participant received two rTMS treatments: one to the right IFJ and one to the left IPS, with site coordinates based on a prior neuroimaging study employing an equivalent decision task (Huettel et al., 2006). Participants also received an active-control session of rTMS to the interhemispheric fissure at vertex, for a total of three rTMS sessions per participant (order of TMS stimulation sites was counter-balanced over participants to control for any order effects). Sessions applied 15 min of 1-Hz rTMS, a protocol shown to disrupt (Harris et al., 2008) or inhibit (Chen et al., 1997) neural information processing for approximately 10 min (Robertson et al., 2003; Eisenegger et al., 2008). Immediately following rTMS, participants completed a run (165 trials) of the decision-making task. There was a resting period of about 10 min after each run to minimize carryover effects, resulting in a total washout period of approximately 30 min prior to each run of the decision task (5 m previous task run + 10 m rest + 15 m current rTMS session).

Upon completion of the experiment, participants were debriefed and questioned about headaches, discomfort, or any other acute side effects (over-the-counter pain medication was available, but all subjects declined). Prior to release, participants were required to pass an evaluation of basic perceptual, short term, and working memory function, awareness of current time and location. Participants were contacted 24 h after the study to check for any experimental side effects (none were reported).

### Decision-making task

Participants made self-paced choices between certain (e.g., 100% chance of $5) and uncertain options. Uncertain options were either risky (e.g., 50% chance of $12) or ambiguous (e.g., “??”% chance of $12). Stimuli and task design were adapted from prior studies of risky/ambiguous choices (Huettel et al., 2006; Stanton et al., 2011). Briefly, gambles were presented side-by-side on a gray background, with position of the risky gamble randomized. Gambles were represented as pie charts (Figure 1B), with the probability of winning money indicated by the blue-shaded pie area, and the complementary probability of winning nothing represented by the remaining area shaded yellow. Certain gambles were thus entirely blue. Ambiguous gambles, by contrast, had their shading hidden beneath a white field featuring a black question mark. Dollar magnitudes available to win were superimposed over each shaded area in white, or, for ambiguous gambles, set outside the hidden circle marker in black. For each trial, a fixation dot (150 ms) was followed by the options. Self-paced choices were indicated using the right hand to press the left or right arrow keys, although if longer than 5000 ms or less than 200 ms, a brief message encouraged faster or slower responses, respectively. A box indicated the chosen gamble for 250 ms. Participants did not observe the outcomes of any gambles or receive any gamble bonus winnings until after the completion of all four task runs. Participants were instructed that the winning probability for ambiguous options was hidden, but could take any value from 0 to 100%.

We manipulated the certain option reward amount ($3–$7), uncertain option reward amount ($2–$98) and the degree of option uncertainty (75%, 50%, 25%, or ??%) to incentivize the uncertain option to various degrees. We combined values on these variables to construct 165 distinct gamble scenarios (45 each for 25%, 50%, and 75% risk; 30 for ambiguity). This set of 165 gambles was repeated for each task run with gamble order randomized, allowing repeated measures comparisons controlling for subject, gamble, and subject-by-gamble effects. The majority of these gambles (128 out of 165) were constructed such that the uncertain option had a higher expected value than the certain option, thereby providing an incentive to choose the uncertain option (17 had equal expected value, and 20 had greater certain expected value). We based the level of these incentives on previous observations of risk and ambiguity aversion in this task, such that stronger incentives tended to be provided for conditions characterized by higher risk/ambiguity aversion. The ratio of the uncertain vs. the certain expected value (assuming ambiguous options to have an expected probability of 0.5) thus ranged from 0.5 to 3.6. The higher end of this range was covered by the ambiguous and 25% gambles to sufficiently reward risk taking, as participants are typically strongly risk-averse to such gambles. By contrast, the 75% gambles covered the lower end of this range, with 50% gambles intermediate to these extremes.

To provide participants an incentive to choose according to their preferences, we explained that for each run of the task (165 gamble trials) we would randomly select one trial, and that at the end of the experiment, we would resolve the gambles from those trials according to their choice, and pay them the winnings from each trial. Participants completed four runs of the task (initial practice run and three runs following rTMS sessions) and were thus paid for a total of four such bonus trials. The average bonus compensation was $38.43 per participant (with a range of $8 to $125).

### Anatomical MRI scan acquisition

Anatomical imaging was conducted on a 3.0 Tesla GE Discovery MR750 system using an eight-channel head coil, conducting a T1-weighted FSPGR scan in the axial plane with a 3D inversion recovery prepared sequence (120 slices, 1 mm slice thickness, 1 × 1 mm in-plane resolution).

### Repetitive transcranial magnetic stimulation (rTMS)

We employed an “off-line” 1-Hz rTMS protocol with the goal of disrupting information processing within the targeted brain regions prior to a series of economic decisions involving uncertainty. This paradigm has been previously shown to inhibit primary motor cortex, reduce signal strength in visual processing, and perturb social and economic decision making (Knoch et al., 2006; Camus et al., 2009; Figner et al., 2010; Baumgartner et al., 2011). Research participants were unaware of both the study hypotheses and the presence of a placebo target condition (vertex stimulation). The experiment thus reflected a single-blind placebo-controlled design. Each participant received three 15-min, 900-pulse trains of 1-Hz rTMS, with each applied over a separate brain region at an intensity of 100% of resting motor threshold using a Magstim Rapid^2^ stimulator with a Magstim Double 70 mm Air Film Coil (The Magstim Company Limited, Whitland, Dyfed, UK). Sessions were separated by ~15 min (5 min for decision task and 10 min of break/setup time). Motor threshold was determined for each participant using electromyographic recording of the dorsal interosseus muscle of the right hand. Following standard procedures (Rossi et al., 2009), motor threshold was defined as the lowest percentage of maximum stimulator output required to evoke at least 5 out of 10 motor-evoked potentials with peak-to-peak amplitude of at least 50 μV. Stimulation was conducted with the coil positioned tangential to the skull, perpendicular to underlying gyral/sulcal brain anatomy, and with the coil head more anterior and coil handle more posterior.

### Neuronavigated rTMS targeting

rTMS was applied to three anatomical locations. Standardized MNI coordinates for the IPS (−36, −57, 50) and IFJ (39, 16, 33) were based on peak group activations associated with risk and ambiguity preferences in a previous fMRI study (Huettel et al., 2006). MNI coordinates for the vertex active control site (0, −28, 90) were determined by selecting the coordinates falling most directly over the interhemispheric fissure at the peak of the standardized MNI brain.

Coordinates were identified for each participant using their structural MRI scan and a neuro-navigated rTMS procedure implemented using the Brainsight suite of tools and software (Rogue Research, Montreal, Canada). Each participant's anatomical MRI image was mapped to MNI standard space based on manual registration landmarks (anterior commissure, posterior commissure, brain size, and edges), allowing rTMS targets defined in MNI coordinates to be translated to each individual's native brain anatomy. Next, we co-registered our participants' cranial features with their anatomical MRI scans, using the left and right intertragal notch, nasion, and tip of nose. This allowed us to target the IPS, IFJ, and vertex consistently within individual participants. Participants were re-registered prior to each rTMS administration to insure accurate administration.

### Dependent and independent measures

The dependent variables of primary interest were choice (selection of the certain or uncertain option) and decision time (ms). We used multilevel logistic regression with a logit link function and binary distribution to analyze choices, and multilevel generalized linear regression with a lognormal distribution and an identity link function to analyze decision times. We also estimated the theoretical impact of rTMS stimulation on the average expected return from participants' choices (i.e., the expected consequences if they had been paid for each trial) by modeling rTMS effects on the expected value of the chosen option for each trial. This approach was selected because its repeated-measures nature paralleled our analysis of choices and RT's, and because our small number of compensated trials (1 per run) precluded any meaningful analysis of rTMS consequences on participant's real take-home bonus pay. Estimates were interpreted as ratios of odds, or converted to relative risk, using the formula Relative Risk = Odds Ratio / (1−P_c_) + (P_c_ × Odds Ratio) where P_c_ is the probability of occurrence in the control condition (Zhang and Yu, 1998).

Independent variables of primary interest included the rTMS treatment condition (vertex, IFJ, or IPS), the difference of uncertain and certain option reward magnitudes (continuous), the uncertain option probability (25, 50, 75%, or ambiguous), and the interactions of these variables. Variables included in our models but not of primary interest were a categorical variable reflecting the rTMS condition order (controlling for any order effects) and variables included to control for any time period effects. For the choice model, we included a categorical fixed-effects time variable reflecting the task run number, while in the decision time model, we included both fixed, and random-effects for a continuous variable reflecting the total number of trials already completed (i.e., controlling for practice effects). These control variables helped account for time effects including a clear practice effects for response times as well as a slight increase in risky choices by the end of the study.

Study personnel responsible for data analysis and modeling were blinded to the rTMS treatment conditions during the primary stages of data analysis, as rTMS conditions were coded as an arbitrary single-digit number. This coding was maintained until after omnibus tests demonstrated significant interaction of (coded) rTMS treatment with uncertainty type. The code blinding was lifted only when it became necessary to test previously hypothesized contrasts between the experimental and control conditions.

### Repeated measures and multilevel modeling

We implemented multilevel mixed-effects models for repeated measures (Snijders, 2011) to account for non-independence due to our design (165 gambles repeated across three rTMS treatments) and subject effects (individual differences in risk/ambiguity aversion and average decision speed). The 165 gamble scenarios were modeled as “subject level” observations (three observations each), while participants were modeled as “group level” observations. We thus controlled for sources of dependency by simultaneously accounting for variance at the trial and participant levels, allowing for valid inferences regarding expected rTMS effects in the broader population. High intraclass correlations (indicating a violation of the independence assumption) for each of our independent variables confirmed that a mixed model approach was justified. This approach also allowed us to address trial-varying effects (i.e., practice effects on decision speed, which evolve through time) and participant-varying effects (e.g., broader preference or personality trait influences on decisions).

We fit models using SAS 9.3 Proc GLIMMIX (Sas Institute, 2011). Models were estimated using residual pseudo-likelihood estimation with subject-specific Taylor series expansion (Breslow and Clayton, 1993). The residual degrees of freedom were determined using the improved F approximation procedure described by Kenward and Roger (2009). To avoid unnecessary statistical comparisons between conditions, we restricted pairwise comparison in two ways. First, we examined only rTMS treatment effects, always matching other model factors across comparisons (i.e., comparing choices on the 50% trials between control, IFJ, and IPS rTMS, but never comparing 50% trials directly to 25% trials). Second, we conducted such comparisons only when the omnibus test for the effect (Type III sum of squares *F*-test) was significant at the 5% level.

An advantage of a multilevel models approach to repeated measures is that missing-at-random observations are permissible. Data was missing for three rTMS sessions in our study: one participant declined to complete the IFJ rTMS condition, while neuro-navigated targeting failed for two rTMS sessions (one IFJ, one vertex).

## Results

**Figure 2:**
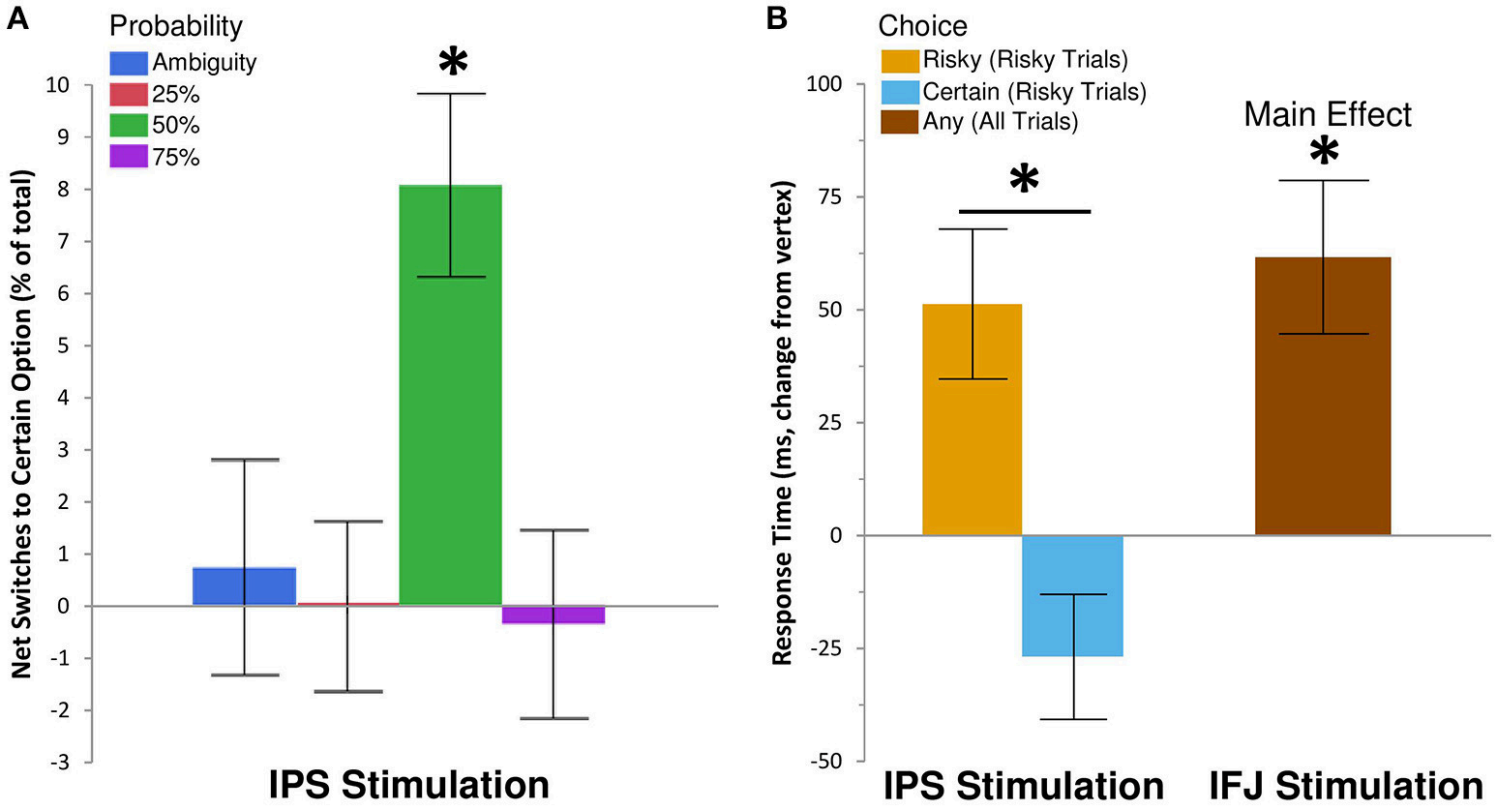
**IPS and IFJ stimulation differentially affect risky decision making**. **(A)** Disruption of IPS using rTMS biased risky choices on 50% probability trials toward certain options relative to matched choices in the vertex rTMS condition. Positive values indicate that switches from risky options (during vertex rTMS) to safe options (during IPS rTMS) exceeded switches in the opposite direction. A null effect of rTMS on choice would show an effect near zero. **(B)** Disruption of IPS biased response times for risky trials, speeding selection of the certain option but slowing selection of the risky option. By contrast, IFJ stimulation slowed decisions across both risky and ambiguous trials, regardless of choice. All bars indicate means ± SE. ^*^P < 0.05.

We examined the effects of rTMS on decision making by recording choice and response time on each gamble trial. Descriptive statistics are shown in Table 1, while inferential tests and degrees of freedom are reported below as Kenward-Rogers approximations, thereby incorporating a conservative adjustment appropriate for mixed, unbalanced designs in the behavioral sciences (Kenward and Roger, 2009). We hypothesized that, compared to control rTMS, perturbation of processing within IPS would reduce risk-taking for risky choices, while disruption of IFJ processing would similarly affect ambiguous choices (cf. Huettel et al., 2006; Bach et al., 2011). Results confirmed our hypothesis for IPS stimulation, which biased choices toward the certain options within the 50% probability condition [*F*_(6, 6905)_ = 2.42, *P* = 0.02; Figure 2A]. For 50% trials—which involve maximal uncertainty—IPS stimulation increased the probability of choosing the certain option by 30% [95% CI [12%, 48%], *t*_(6905)_ = 3.26, *P* = 0.0011]. This increase in certain choices for IPS stimulation was also significant when compared to the effects of IFJ stimulation on 50% trials [*t*_(6905)_ = 2.62, *P* = 0.0089]. The specificity of this effect to the 50% probability trials is consistent with previous decision making results showing maximal rTMS effects at intermediate choice probabilities (Figner et al., 2010). IFJ stimulation, by contrast, produced no reliable effect on choices (all *P*s 0.09–0.56, Supplemental Results: Choices).

**Table 1:** **Descriptive statistics for choices and response times**.

**rTMS Location**	**Ambiguous**	**25%**	**50%**	**75%**	**All trials**
**PERCENT CHOICE OF UNCERTAIN OPTION BY TRIAL TYPE**					
Vertex	40.8%	43.5%	53.1%	57.8%	49.5%
IPS	40.0%	43.5%	44.3%	58.1%	47.1%
IFJ	42.2%	42.6%	48.3%	55.9%	47.7%
All locations	41.0%	43.2%	48.6%	57.3%	48.1%
**MEAN RESPONSE TIME AND STANDARD DEVIATION (MS) BY TRIAL TYPE**					
Vertex	1088 (438)	1109 (477)	1001 (321)	1049 (404)	1059 (414)
IPS	1097 (506)	1097 (532)	1040 (532)	1050 (516)	1069 (524)
IFJ	1132 (629)	1101 (553)	1032 (457)	1066 (518)	1079 (536)
All locations	1106 (531)	1102 (522)	1025 (446)	1055 (482)	1069 (494)

*Descriptive statistics reflect raw, model-unadjusted effects in participants completing all study conditions. Descriptive statistics reported are distinct from inferential, model-adjusted statistics reported in the manuscript text*.

Since the risky option was incentivized for most of our gambles, more conservative decision making would be expected to reduce uncertainty, but also to reduce expected earnings. To quantify the impact of IPS stimulation on expected earnings (i.e., average theoretical earnings if all gamble decisions were resolved) we analyzed a model in which the dependent variable was the expected value of the chosen option (with ambiguous probability modeled as 0.50). We examined the difference in expected value for the chosen option on 50% probability trials, comparing gambles after IPS stimulation to matched gambles after vertex control stimulation. The results of this comparison revealed that the 30% increase in certain choices induced by IPS stimulation corresponded to a 5% decrease in average expected value relative to control rTMS [-$0.34 per trial/-$15.30 per subject, *t*_(4119)_ = −4.1, *p* = 0.0001, Supplemental Results: Expected Value]. IPS stimulation thus affected choices such that both risk-taking and expected rewards were reduced for 50% probability trials.

To gain further insight into the observed decrease in risk-taking following IPS stimulation, we examined response time (RT), which is often better suited to revealing subtle influences of rTMS on the efficiency of information processing (Luber and Lisanby, 2014). The effects of rTMS location on RT for the 25, 50, and 75% probabilities were similar in magnitude and direction [interaction *F*_(4, 5637)_ = 0.6, *P* = 0.66], so we collapsed these trials into a single “risky decision” category. Our subsequent analysis showed a main effect of rTMS location on RT [*F*_(2, 4676)_ = 7.18, *P* = 0.0008], which was qualified by a three-way interaction of rTMS location, trial type (risky or ambiguous), and chosen option [certain or uncertain; *F*_(2, 6895)_ = 3.81, *P* = 0.02]. IPS stimulation did not affect RTs for ambiguous trials, but did so on risky trials, for which the effect was moderated by the chosen outcome [*t*_(6892)_ = 4.01, *P* = 0.0001; Figure 2B]. Specifically, IPS stimulation slowed choices of the uncertain option [Δ RT = 52 ms, +5.64%, 95% CI [2.11%, 9.29%], *t*_(5116)_ = 3.16, *P* = 0.002], but trended toward *facilitating* or speeding up choices of the certain option [Δ RT = −27 ms, −2.88%, 95% CI [−5.76%, 0.09%], *t*_(4421)_ = −1.9, *P* = 0.057]. No other contrasts showed evidence of decision speeds faster than their corresponding vertex control (Supplemental Results: Response Times). By comparison, IFJ stimulation resulted in a general disruption of decision processing slowing choices across all trial types [Δ RT = 52 ms, +5.59%, 95% CI [2.61%, 8.66%], *t*_(5148)_ = 3.73, *P* = 0.0002; Figure 2B].

## Discussion

Effective decision makers are adept at weighing the potential benefits of an opportunity against the uncertainty surrounding their realization. Here, we examined the contributions of the left IPS and right IFJ to such decision making by manipulating neural activity within these regions using 1-Hz rTMS. IPS stimulation reduced risk-taking on risky decision trials, while IFJ stimulation slowed decision responses across both risky and ambiguous decision trials. These results provide the first causal evidence differentiating parietal and frontal contributions to risky decision making, and highlight the IPS as a key region supporting the expression of risk-tolerant choices.

In this study, disrupting IPS activity reduced risk-taking—lowering risk at a cost to expected earnings—for decisions with high but known risks and uncertain outcomes. These results demonstrate a causal role for the IPS in risky decision making that is in line with correlative evidence from previous neuroimaging studies. These fMRI studies demonstrated a positive association between IPS activation and increased risk-taking (Huettel et al., 2006; Bach et al., 2011), with IPS activity preferentially tracking the subjective value of risky (as opposed to delayed) decision options (Peters and Büchel, 2009). Our study, which estimated rTMS treatment effects on decisions by repeating gambles within-subjects, compliments prior methods by applying rTMS with strong experimental controls for individual differences. The results of our stimulation study buttress the existing evidence by providing the first causal evidence for the necessity of IPS function for pursuing risky decisions.

Given previous results linking IPS activation to individual differences in risk preferences (Huettel et al., 2006), we interpret IPS stimulation as biasing risk (but not ambiguity) preferences toward certainty; this slows selection of risky options, speeds selection of certain options (Figure 2B) and reduces risk-taking behavior (Figure 2A). Our results confirm the importance of the IPS for risky decision making, but provide only indirect evidence for how computations within that region were disrupted. One possibility is that parietal stimulation interfered with an IPS-supported representation of outcome uncertainty, which is at a maximum for the 50% trials most affected by rTMS in our study. Evidence suggesting that the IPS directly represents probability or outcome uncertainty is lacking, however (Tobler et al., 2007; Smith et al., 2009). Rather, IPS appears to represent higher-order decision quantities reflecting the integration of probabilistic information with other reward and preference signals (Huettel et al., 2005, 2006; Peters and Büchel, 2009). Similarly, studies of perceptual decision making in non-human primates indicate that this area of parietal cortex is essential for the integration of evidence in favor of competing choices (Shadlen and Newsome, 2001; Huk and Shadlen, 2005). This mechanism has been extended to value-based decision making, with electrophysiological (Kiani and Shadlen, 2009) and human neuroimaging (Basten et al., 2010) results converging on a role for the IPS in integrating and accumulating the net costs and benefits required to flexibly direct choices. Consistent with this putative mechanism, we observed that inhibition of IPS led to faster selection of the competing certain option but slower selection of the risky option. In such a model, direct effects of parietal rTMS on choice would be most likely for balanced decisions featuring little accumulation of net evidence toward either option—such as our 50% risk trials—particularly since such dilemmas produce weak neuronal responses vulnerable to exogenous disruption through rTMS (Kiani and Shadlen, 2009). IPS stimulation may thus directly impact uncertain decision processing by disrupting the integration of probabilistic costs and benefits required to justify a risky choice.

The IPS is also known as a key locus within a network supporting numerical cognition (Cohen Kadosh et al., 2007; Piazza et al., 2007; Dehaene and Brannon, 2011). A natural supposition, therefore, is that IPS stimulation disrupts processes necessary for quantifying costs and benefits during risky decision making, such as expected value computation or risk-discounted value comparison. While our experiment was not designed to dissociate such processes, we again note that IPS stimulation affected risk-taking only for the 50% probability condition, and not for the 25%, and 75% conditions. In our study, value computations were simplest for 50% probabilities, while value *comparisons* were more demanding, since the model-adjusted baseline (i.e., vertex TMS control) percentage of risky choices for these trials was 60%, indicating that differences in risky and certain subjective values were small compared to the other probability conditions (model adjusted baseline values for other conditions were 21%, 90%, and 28% for the 25% risk, 75% risk, and ambiguity conditions, respectively). Prior neuroeconomic findings suggest that the often-subtle disruptions of processing induced by rTMS are most clearly revealed in overt behavior at such “tipping points,” where subjective preferences are close to indifference (Figner et al., 2010). Additionally, IPS activation has been shown to parametrically represent the closeness of numeric magnitudes (Ansari et al., 2006). Given this pattern of results, we speculate that rTMS may have disrupted relative value comparison processes supported by the IPS (Dorris and Glimcher, 2004), as opposed to altering the direct computation of risky expected values. Decreased confidence in the relative premium offered by the risky option could thereby reduce willingness to accept those options.

Despite previous evidence linking IFJ activation with ambiguity preferences (Huettel et al., 2006; Bach et al., 2011), our RT results support a largely general role for IFJ in decision making under uncertainty, with both risky and ambiguous choices slowed by IFJ stimulation. Further studies integrating executive and motor control tasks are required to determine whether this slowing results from interference with higher-order decision-control or lower-order decision-implementation processes. Recent findings suggest that although IFJ is engaged by the presence of ambiguity, its activity does not scale with increasing degrees of ambiguity (Bach et al., 2011; Bach and Dolan, 2012), raising questions regarding the interpretation of previous findings featuring categorical—rather than continuous—measures of ambiguity (Huettel et al., 2006; Bach et al., 2009). Instead, IFJ engagement during ambiguous choice may reflect the increased cognitive control required to address problems featuring missing or hidden information (Koechlin et al., 2003; Brass and von Cramon, 2004; Bach et al., 2011; Helfinstein et al., 2014). Ambiguous decisions may be implemented in qualitatively different ways less reliant on numerical processing or IPS-mediated magnitude comparison (Camerer and Weber, 1992; Bach and Dolan, 2012), as we found no clear evidence supporting an effect of IPS stimulation on ambiguous choices. Conclusions regarding the specific roles of IFJ and IPS during ambiguous decision making remain speculative, however. Variable strategic responses and a relatively low trial-level sample size for ambiguous gambles may have limited our power to identify effects.

Our investigation relied on the ability of 1 Hz rTMS to induce short-term neurophysiological changes in target brain regions, thereby disrupting typical cognitive processing. Though this effect permits causal investigations of neuroanatomical hypotheses, it also imposes limitations on the interpretation of our within-subject design study, as residual effects from earlier stimulation sessions have the potential to carry over to subsequent sessions. Consideration of the time course of these effects is of particular importance for our design, since we conducted three consecutive rTMS sessions per subject. Previous evidence from behavioral and simultaneous PET/TMS studies suggests that 15 min of 1 Hz rTMS should influence behavior and alter regional cerebral blood flow for about 5–15 min (Chen et al., 1997; Lewald et al., 2002; Mottaghy et al., 2002; Eisenegger et al., 2008). In our study, we sought to limit the carryover of behavioral influences from prior rTMS sessions by imposing a break period between sessions. Approximately 30 min separated task runs from previous rTMS sessions, a period which is 2–3 times longer than the expected time required for behavioral effects of rTMS to dissipate.

However, while behavioral effects of our rTMS stimulation sessions were expected to dissipate within 15 min, prior studies have found subtle electrophysiological after-effects of 1 Hz rTMS up to about 40 min post-stimulation, even in the absence of behavioral effects (as reviewed by Rossi et al., 2009). Research into the neurophysiological basis of such extended rTMS effects is ongoing (Hamidi et al., 2009; Thut and Pascual-Leone, 2010; Noh et al., 2012; Chung et al., 2015), and future studies should seek to specifically characterize the electrophysiological profile of repeated rTMS sessions. Within the design and analysis approach of the present study, we attempted to mitigate against these potential longer-term carryover effects via two additional counter-measures. First, we counterbalanced the order of rTMS sessions, such that any carryover effects should in theory be similarly affecting the different stimulation conditions. Second, we also included the sequence order of rTMS sessions as a control predictor in our statistical models (Supplemental Results, Tables S1 and S6), such that any effects of stimulation order should in principle be accounted for in the results. Nevertheless, given the inherent susceptibility of a within-subjects design to carryover effects, it would be beneficial for independent researchers to corroborate the present findings using convergent methods, including between-subjects designs and alternative rTMS protocols such as 5 Hz or theta-burst stimulation (Peinemann et al., 2004; Huang et al., 2005; Zafar et al., 2008).

Our results are the first to demonstrate the necessity of unperturbed IPS function for risk tolerance during uncertain decision making, and provide insight into the functions and interactions of fronto-parietal decision circuits. Our focus on the parietal cortex during risky decision making also complements prior rTMS work showing *increased* risk-taking and impulsivity following disruption of prefrontal self-control processes (Knoch et al., 2006; Figner et al., 2010). Future work could extend our findings using between-subjects designs amenable to the investigation of individual differences in risk preferences, as well as by probing the extent of IPS effects by continuously varying probability around the 50% level noted here. The present findings suggest that engagement of IPS during decision making may support the ability to trade certainty for a chance at greater expected gains.

## Author contributions

CC, SH, and TE designed the experiment; CC, AK, and FK administered the study; CC analyzed the data; CC, SH, and TE wrote the manuscript; CC, TE, SH, AK, and FK edited and approved the manuscript.

### Conflict of interest statement

The authors declare that the research was conducted in the absence of any commercial or financial relationships that could be construed as a potential conflict of interest.

## Abbreviations

IPS: Intraparietal sulcus
IFJ: inferior frontal junction
rTMS: repetitive Transcranial Magnetic Stimulation.
