# Supplementary material for: Reduced Risk-Taking following Disruption of the Intraparietal Sulcus
Source: Front Neurosci. 2016 Dec 23;10:588. doi: 10.3389/fnins.2016.00588 (PMC5179562; doi:10.3389/fnins.2016.00588)
Supplement: Supplementary file 1 [file DataSheet1.PDF]

Supplementary Material

Reduced Risk-Taking Following Disruption of the Intraparietal Sulcus

Christopher G. Coutlee[1], Anastasia Kiyonaga[1], Franziska M. Korb[1], Scott A. Huettel[1], Tobias Egner[1*]

[1]Center for Cognitive Neuroscience, Department of Psychology and Neuroscience, Duke University, Durham, NC, USA

*** Correspondence:**
Tobias Egner
tobias.egner@duke.edu

Supplementary results

Choices

Choices were fit using a generalized linear mixed logistic regression using a binary distribution and a logit link function. Estimates were interpreted as ratios of odds, or converted to relative risk, using the formula Relative Risk = Odds Ratio / (1 – P_c) + (P_c x Odds Ratio) where P_c is the probability of occurrence in the control condition. Results from this model are depicted below.

Table S1

Multilevel Logistic Regression Model: Choices

**Omnibus Test of Fixed Effects (Type III)**	**Num DF**	**Denom DF**	***F***	***p***
rTMS Condition Order (control variable, categorical)	5	2422	117.99	<.0001
Number of Task Runs (time effects, categorical)	2	6905	5.12	0.006
Difference of Option Reward Amounts (continuous)	1	2821	393.25	<.0001
Trial Probability (categorical)	3	2625	100.80	<.0001
Reward Difference x Probability	3	2576	23.22	<.0001
rTMS Treatment Condition (categorical)	2	6905	1.84	0.16
rTMS Condition x Reward Difference	2	6905	0.77	0.47
rTms Condition x Probability	6	6905	2.42	**0.02**
**Random Effects**	**Estimate**	**SE**		
Paired-trial (Within-subject) Variance	1.957	0.124		
Model Deviance	33023			

Table S2

Interaction of rTMS Condition and Gamble Probability, Contrasts of Model-Estimated Means (Least Squares Means)

rTMS_1	Prob_1	rTMS_2	Prob_2	Estimate	Odds Ratio	SE	DF	*t*	*p*
IPS	Ambiguity	Vertex	Ambiguity	-0.028	0.972	0.1916	6905	-0.15	0.88
IPS	0.25	Vertex	0.25	-0.1980	0.820	0.1737	6905	-1.14	0.25
IPS	0.75	Vertex	0.75	0.04107	1.042	0.1610	6905	0.26	0.80
IPS	0.5	Vertex	0.5	0.4859	1.63	0.1490	6905	3.26	**0.0011**
IFJ	Ambiguity	Vertex	Ambiguity	-0.3274	0.721	0.1950	6905	-1.68	0.09
IFJ	0.25	Vertex	0.25	-0.2413	0.786	0.1782	6905	-1.35	0.18
IFJ	0.75	Vertex	0.75	0.1606	1.174	0.1659	6905	0.97	0.33
IFJ	0.5	Vertex	0.5	0.08891	1.093	0.1531	6905	0.58	0.56
IPS	Ambiguity	IFJ	Ambiguity	0.2994	1.349	0.1919	6905	1.56	0.12
IPS	0.25	IFJ	0.25	0.0434	1.044	0.1747	6905	0.25	0.80
IPS	0.75	IFJ	0.75	-0.1195	0.887	0.1619	6905	-0.74	0.46
IPS	0.5	IFJ	0.5	0.3970	1.487	0.1516	6905	2.62	**0.0089**

Figure S1. IPS stimulation biases choices during 50% gambles. Choices after IFJ stimulation, as well as choices after IPS stimulation for non-50% gamble probabilities, could not be distinguished from choices during corresponding vertex control stimulation. Odds ratio is plotted on a logarithmic scale. A value of 1 reflects no change in the odds of choosing the certain option relative to vertex stimulation. All bars indicate estimate ± standard error.

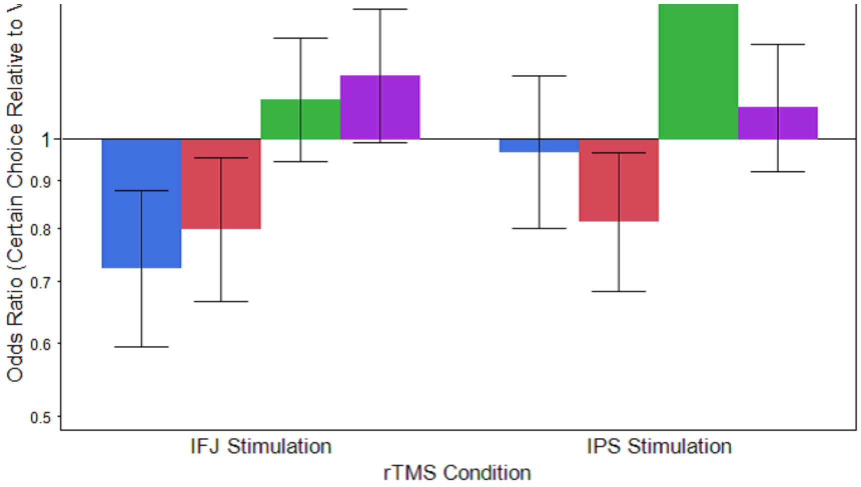

Figure S1. IPS stimulation biases choices during 50% gambles. Choices during IFJ stimulation, and of IPS stimulation for all other gamble probabilities, could not be distinguished from choices during corresponding vertex control stimulation. Odds ratio is plotted on a logarithmic scale. A value of 1 reflects no change in the odds of choosing the certain option relative to vertex stimulation. All bars indicate estimate ± standard error.

To aid in the interpretation of the choice results, we also calculated switches for each matched decision trial (compared with choice on the matched trial in the vertex rTMS condition) after IPS and IFJ rTMS. We plotted the net number of switches towards certainty (i.e. safe switches-risky

switches) as a percentage of the total number of possible switches for the significant IPS stimulation condition (Figure 2a).

Table S3
Trial Count Totals for Choice Switching vs Vertex Choice (Figure 2a)

rTMS	Probability	No Switch	Risky Switch	Safe Switch	Net Safe Switch	% Net Safe Switch
IPS	Ambiguity	353	32	35	3	0.71
IPS	0.25	536	47	47	0	0.00
IPS	0.75	514	59	57	-2	-0.32
IPS	0.5	518	32	80	48	7.62
IFJ	Ambiguity	305	30	25	-5	-1.39
IFJ	0.25	443	46	51	5	0.93
IFJ	0.75	436	47	57	10	1.85
IFJ	0.5	432	41	67	26	4.81

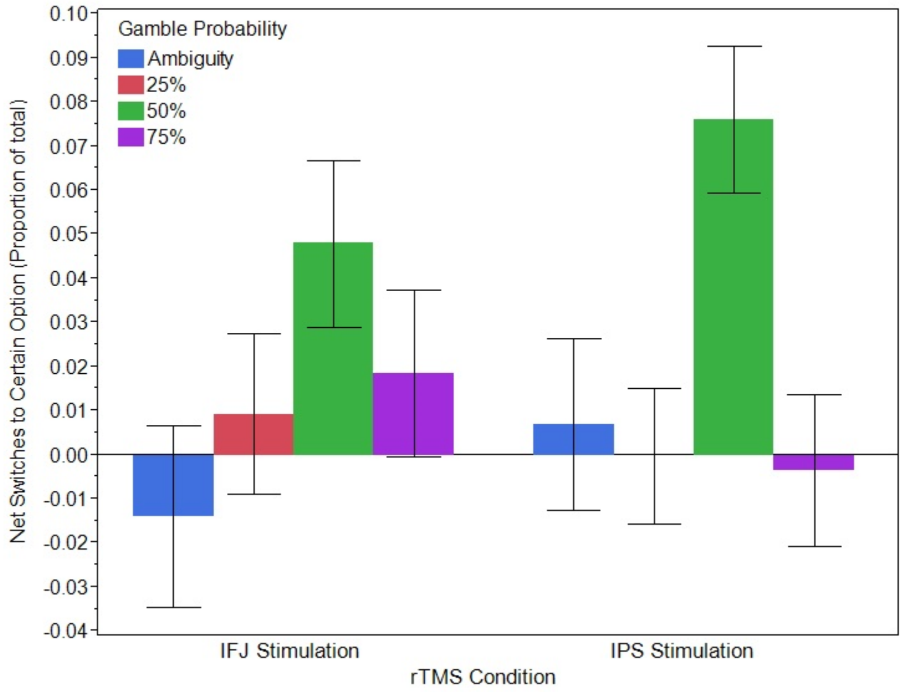

Figure S2. Trial count totals for choice switching relative to vertex choices for IFJ and IPS stimulation. Inhibition of IPS using rTMS biased risky choices on 50% probability trials towards the safer but less profitable certain option, relative to matched choices in the vertex rTMS condition. All bars indicate estimate ± standard error.

choices, we analyzed a model with the normative expected value of the chosen option (ambiguity =

50%) as the dependent variable. Expected values were log-normally distributed, leading us to fit a multilevel generalized linear mixed model (as described for response time). Estimates were back-transformed to dollars by raising the constant *e* to the power of the parameter estimate (i.e., the exponential, or inverse natural log function, e^{Estimate}). Our interest was on the difference in the expected value of chosen options in the 50% probability trials after IPS stimulation, relative to vertex stimulation. For this comparison of interest, results below showed a $0.34 difference in expected value per decision. Across the 45 50% trials in the IPS rTMS run, this equals an expected value difference of $15.30.

Table S4
Multilevel Generalized Linear Model: Expected Value of Chosen Option

Omnibus Test of Fixed Effects (Type III)	Num DF	Denom DF	*F*	*p*
rTMS Treatment Condition (categorical)	2	4202	3.2	0.041
Trial Probability (categorical)	3	2398	53.41	<.0001
rTms Condition x Probability	6	4177	2.48	**0.0216**
**Random Effects**	**Estimat e**	**SE**		
Paired-trial (Repeated Measures) Variance	0.2263	0.0071		
Residual Decision-level Variance	0.05.4	0.0011		
Model Deviance	5177			

Table S5
Interaction of rTMS Condition and Probability on Expected Value of Chosen Options, Contrasts of Model-Estimated Means (Least Squares Means)

rTMS_1	Prob_1	rTMS_2	Prob_2	Estimate	SE	DF	*t*	*p*	Estimate (Dollars)
IPS	0.5	Vertex	0.5	-0.05137	0.01254	4119	-4.1	**<.0001**	**0.34**

Response times

To reveal any influences of rTMS on decision processing which were not apparent in overt choices, we chose to analyze response times on decision trials. After fitting an initial model response time residuals appeared log-normally distributed, so to more appropriately model this outcome, we fit a generalized linear mixed model using a log-normal distribution with an identity link function. Estimates were back-transformed to milliseconds by raising the constant *e* to the power of the parameter estimate (i.e., the exponential, or inverse natural log function, e^{Estimate}). In contrast to our choice models, the major source of repeated-measures correlation in the RT data was speeding of

response over time (practice effects), rather than correlations between matched, repeated gambles across runs. Because each subject performed a full (paid) run of 165 trials (not analyzed here) prior to receiving any rTMS, the practice effects observed in our data were relatively linear, although their slope did differ from subject to subject. To account for this variance, we included three random effects: a random subject level intercept, a random slope for the effect of practice (number of trials completed) for each subject, and a covariance term between this slope and the intercept. The inclusion of personalized regression estimates of practice effects substantially improved model fit (although the variance estimate for the practice effect slope itself was small).

IFJ stimulation produced a main effect of rTMS, but we failed to find sufficient evidence to interpret an interaction beyond this main effect for IFJ, as all conditions showed a similar pattern of slowing relative to vertex. Instead, the overall three way interaction of rTMS condition, Choice, and Trial Type appeared to be driven by the effects of IPS stimulation on response times (relative to vertex), which were moderated by choice. These effects for IFJ and IPS stimulation are illustrated below and in Figure 2b.

Table S6
Multilevel Generalized Linear Model: Response Time

**Omnibus Test of Fixed Effects (Type III)**	**Num DF**	**Denom DF**	***F***	***p***
rTMS Condition Order (control variable, categorical)	1	13.01	1.85	0.20
Number of Trials Completed (time effects, continuous)	1	13.51	26.69	0.0002
Choice (certain or uncertain option, categorical)	1	6898	28.25	<.0001
Trial Type (risky or ambiguous, categorical)	1	6889	15.98	<.0001
rTMS Treatment Condition (categorical)	2	4676	7.18	**0.0008**
Choice x Trial Type	1	6891	36.28	<.0001
rTMS Condition x Choice	2	6880	1.82	0.16
rTMS Condition x Trial Type	2	6890	0.92	0.40
rTMS Condition x Choice x Trial Type	2	6895	3.81	**0.0221**
**Random Effects**	**Estimate**	**SE**		
Subject Intercept	0.0917	0.0361		
Slope of Time/Practice Effect per Subject	0.000027	0.000042		
Intercept/Slope Covariance	~0	Undefined		
Residual Decision-level Variance	0.0882	0.0015		
Model Deviance	3066			

Table S7

Main Effects of rTMS Condition on Response Time, Contrasts of Model-Estimated Means (Least Squares Means)

rTMS_1	rTMS_2	Estimate	SE	DF	*t*	*p*	Estimate (ms)
IPS	Vertex	0.01857	0.01439	4285	1.29	0.20	18
IFJ	Vertex	0.0544	0.01459	5148	3.73	**0.0002**	**52**
IPS	IFJ	-0.03583	0.01444	4690	-2.48	**0.0131**	**-35**

Table S8

Interaction of rTMS Condition, Trial Type, and Choice, Contrasts of Model-Estimated Means (Least Squares Means)

rTMS_1	Choice_1	rTMS_2	Choice_2	Trial Type	Estimate	SE	DF	*t*	*p*	Effect (ms)
IPS	Uncertain	IPS	Certain	Risky	0.05916	0.01475	6892	4.01	**<.0001**	**78**
IPS	Uncertain	Vertex	Uncertain	Risky	0.05482	0.01733	5116	3.16	**0.002**	**52**
IPS	Certain	Vertex	Certain	Ambig	0.02322	0.02657	6518	0.87	0.38	21
IPS	Uncertain	Vertex	Uncertain	Ambig	0.02549	0.03543	6838	0.72	0.47	26
IPS	Certain	Vertex	Certain	Risky	-0.02924	0.01537	4421	-1.9	**0.057**	**-27**

Supplementary methods

Experimental design

rTMS stimulation sessions, decision task runs, and washout rest periods were interspaced as depicted in Figure S3 below (rTMS targets were counterbalanced, such that condition order varied for each subject).

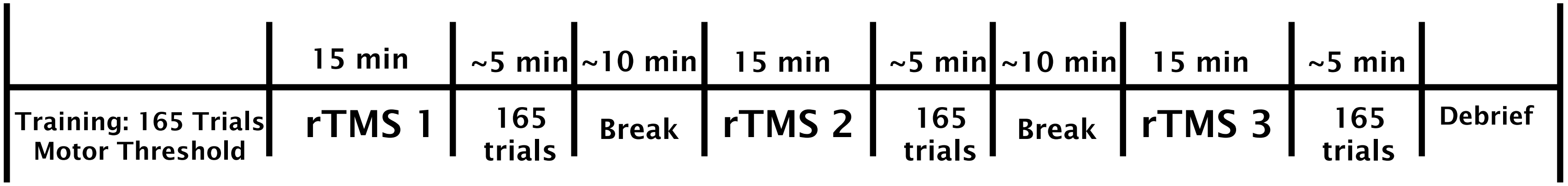

Figure S3. Experimental design. rTMS stimulation sessions were each immediately followed by a run of the decision task. Washout break periods followed each run of the task. This sequence was repeated three times for each study, in order to stimulate the IPS, IFJ, and vertex. Time estimates are approximate because the lengths of the decision task and rest/TMS setup periods were self-paced or variable.
